# Characterization, sub-cellular localization and expression profiling of the isoprenylcysteine methylesterase gene family in *Arabidopsis thaliana*

**DOI:** 10.1186/1471-2229-10-212

**Published:** 2010-09-27

**Authors:** Ping Lan, Wenfeng Li, Huizhong Wang, Wujun Ma

**Affiliations:** 1 State Agricultural Biotechnology Centre, Murdoch University; & Western Australian Department of Agriculture & Food; Murdoch, WA 6150, Australia; 2 College of Life and Environmental Sciences, Hangzhou Normal University, Hangzhou, 310036, China

## Abstract

**Background:**

Isoprenylcysteine methylesterases (ICME) demethylate prenylated protein in eukaryotic cell. Until now, knowledge about their molecular information, localization and expression pattern is largely unavailable in plant species. One ICME in Arabidopsis, encoded by At5g15860, has been identified recently. Over-expression of At5g15860 caused an ABA hypersensitive phenotype in transgenic Arabidopsis plants, indicating that it functions as a positive regulator of ABA signaling. Moreover, ABA induced the expression of this gene in Arabidopsis seedlings. The current study extends these findings by examining the sub-cellular localization, expression profiling, and physiological functions of ICME and two other ICME-like proteins, ICME-LIKE1 and ICME-LIKE2, which were encoded by two related genes At1g26120 and At3g02410, respectively.

**Results:**

Bioinformatics investigations showed that the ICME and other two ICME-like homologs comprise a small subfamily of carboxylesterase (EC 3.1.1.1) in Arabidopsis. Sub-cellular localization of GFP tagged ICME and its homologs showed that the ICME and ICME-like proteins are intramembrane proteins predominantly localizing in the endoplasmic reticulum (ER) and Golgi apparatus. Semi-quantitative and real-time quantitative PCR revealed that the *ICME *and *ICME*-like genes are expressed in all examined tissues, including roots, rosette leaves, cauline leaves, stems, flowers, and siliques, with differential expression levels. Within the gene family, the base transcript abundance of *ICME-LIKE2 *gene is very low with higher expression in reproductive organs (flowers and siliques). Time-course analysis uncovered that both *ICME *and *ICME*-like genes are up-regulated by mannitol, NaCl and ABA treatment, with *ICME *showing the highest level of up-regulation by these treatments. Heat stress resulted in up-regulation of the *ICME *gene significantly but down-regulation of the *ICME-LIKE1 *and *ICME-LIKE2 *genes. Cold and dehydration stimuli led to no significant change of both *ICME *and *ICME*-like gene expression. Mutant *icme-like2-1 *showed increased sensitivity to ABA but slightly decreased sensitivity to salt and osmotic stresses during seed germination.

**Conclusions:**

It is concluded that the ICME family is involved in stress and ABA signaling in Arabidopsis, probably through mediating the process of demethylating prenylated proteins. Identification of these prenylated proteins will help to better understand the significance of protein prenylation in *Planta*.

## Background

Prenylation is a stable lipid modification process involving covalent addition of either farnesyl (15-carbon) or geranylgeranyl (20-carbon) isoprenoids to conserved cysteine residues at or near the C-terminus of proteins [1-3]. It is believed that about 2% of eukaryotic cell proteins are modified by prenylation [2], which is accomplished by three distinct heterodimeric protein isoprenyltransferases. Protein with the carboxyl-terminal residue of the CaaX motif, where "a" refers to the aliphatic residue, is recognized either by farnesyltransferase (FTase) when "X" is methionine, glutamine, serine, alanine, or cysteine, or by geranylgeranytransferase type I (GGTase I) when "X" is leucine or isoleucine [1-3]. FTase and GGTase I are cytosolic enzymes utilizing farnesyl pyrophosphate and geranylgeranyl pyrophosphate as the isoprenyl donors, respectively [1-3]. The third isoprenyltransferase is geranylgeranytransferase type II (GGTase II), which transfers two geranylgeranyl groups from geranylgeranyl diphosphate to the carboxyl terminal cysteine residues of XCCXX, XXCXC, XXCCX, XXXCC, XCXXX or CCXXX motifs [4]. All three enzymes have been found in eukaryotes including protozoans, metazoans, fungi, and plants. In the plant kingdom, these enzymes have been reported in different species such as pea (*Pisum sativum*) [5,6], tomato (*Solanum lycopersicum*) [7,8], and *Arabidopsis thaliana *[9-13]. In Arabidopsis, FTase and GGTase I share a common α-subunit encoded by a single gene *PLP *(PLURIPETALA) [12], whereas the genes encoding the β-subunit of FTase and GGTase I are *ERA1 (ENHANCED RESPONSE TO ABA1) *[9,10] and *GGB (GERANYLGERANYLTRANSFERASE BETA) *[11,13], respectively.

Following prenylation by FTase or GGTase I in cytoplasm, proteins are usually subject to further maturation processing in the endoplasmic reticulum (ER), including cleavage of the 'aaX' residues by endoproteases and methylation of the newly created carboxyl terminal residue cysteine by isoprenylcycteine methyltransferase (ICMT) [1-3]. In Arabidopsis, two genes encoding CaaX endoprotease, AtSTE24 (At4 g01320) and AtFACE-2 (At2g36305 or *AtRCE1*), have been identified [14-16]. Similarly, two ICMT genes, AtSTE14A (At5g23320 or *AtICMTA*) and AtSTE14B (At5g08335 or *AtICMTB*), have also been characterized [17-19]. Using S-adenosyl-L-methionine as a methyl donor, AtSTE14A and AtSTE14B catalyze the methylation of biologically relevant isoprenylcysteine substrates, i.e. farnesylcysteine and geranylgeranylcysteine, but not geranylcysteine [17-19].

Biochemically, protein prenylation and subsequent mature processing steps increase its C-terminal hydrophobicity, which facilitate its attachment to membrane and, in some cases, promoting protein-protein interactions [1-3]. Physiologically, these protein lipid modifications including prenylation and subsequent methylation exert profound effects on diverse processes involving signal transduction and intracellular trafficking pathways [1-3]. In Arabidopsis, protein isoprenylation and its processing steps are involved in hormone metabolism and signaling, such as cytokinin biosynthesis, abscisic acid (ABA) and auxin signaling, meristem development, innate immunity, and other fundamental processes [4].

As protein function can be modulated by phosphorylation and dephosphorylation, it is believed that methylation of isoprenlated protein can be reversible by isoprenylcysteine methylesterase (ICME), and only this step can be reversible during protein prenylation and processing [1,2]. Indirect evidence showed that those cell membranes that can methylate prenylated protein are also capable of demethylating prenylated amino acids N-acetyl-S-farnesyl-L-cysteine [20]. Recently, Deem et al. [21] identified an ICME coding gene At5g15860 in Arabidopsis. Two related Arabidopsis genes, At1g26120 and At3g02410, were also reported in their study.

According to the latest version of TAIR9 (The Arabidopsis Information Resource) released at June 19, 2009, there are two At3g02410 splice variants, 1269 and 1062 bases, encoding two distinct protein products http://www.arabidopsis.org. The 1269 base At3g02410.1 transcript is predicted to encode a 422 amino acid polypeptide while the 1062 base At3g02410.2 transcript is predicted to encode a 353 amino acid polypeptide. Proteins encoded by both splice variants are different from the one reported by Deem et al [21] in which At3g02410 was predicted to encode a 373 amino acid with no predicted trans-membrane domain. The At1g26120 and At5g15860 data in TAIR9 is consistent with Deem et al [21], which highlights two At5g15860 splice variants encoding two distinct protein products and one single At1g26120 splice form http://www.arabidopsis.org.

The ICME activity was reported in Arabidopsis membrane fractions [21], although the precise sub-cellular localization of this protein remains unknown. Overexpressing ICME in Arabidopsis resulted in an ABA hypersensitive phenotype in stomatal closure and seed germination, indicating ICME is a positive regulator of ABA signaling. Furthermore, the expression of this ICME gene can be induced by ABA after 24 hr treatment [22]. Despite these, knowledge about the protein's sub-cellular localization and expression patterns remains unavailable. Moreover, studies on this gene in response to other abiotic stresses have not been conducted.

In the present study, the ICME and two ICME-like proteins, ICME-LIKE1 and ICME-LIKE2 encoded by respective At1g26120 and At3g02410, were characterized including their sub-cellular localization, tissue-specific expression patterns and responses to different abiotic stresses and ABA. The biological function of ICME-LIKE2 was explored by T-DNA knock out mutant.

## Results

### Characterization and clustering analysis of *ICME *gene family in Arabidopsis

**Figure 1:**
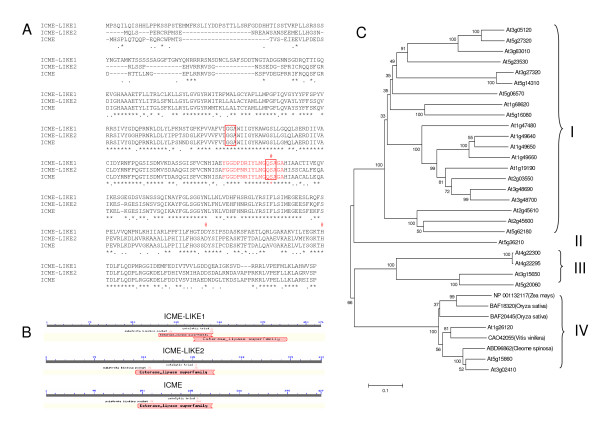
**Characterization of ICME and its homologs**. **(A) **Multiple amino acid sequence alignment of ICME and its homologs was performed using Clustal W multiple alignment program. * represents the residues in that column are identical, : represents that conserved substitution have been observed, and · represents that semi-conserved substitution have been observed. Amino acids marked with red represent the putative carboxylesterase type-B serine active site, amino acids in the boxes represent the substrate binding pocket, and amino acids marked with # represent the catalytic triad. **(B) **Both ICME and its homologs belong to esterase lipase superfamily with conserved domain of carboxylesterase. **(C) **Phylogenetic relationships of ICME and its homologs, their homologs from Zea mays, *Oryza sativa, Vitis vinifera*, and *Cleome spinosa *as well as carboxylesterases from Arabidopsis. The figure showed an unrooted, bootstrap consensus plot, generated by the Neighbor-Joining method using MEGA4 program. The bootstrap values displayed are calculated based on 1000 replications.

Since the gene product of At5g15860 was identified as ICME [21], we named the gene products of At1g26120 and At3g02410 as ICME-LIKE1 and ICME-LIKE2, respectively. To confirm the coding sequences deposited in public databases, the open reading frame (ORF) of these genes were obtained by RT-PCR. We successfully determined a 1431 base ORF for At1g26120, a 1269 base and a 1284 base ORFs for respective At3g02410 and At5g15860. The amplified PCR products were cloned into pGEMT-easy vector and sequenced. Sequencing results showed the ORF sequences of three genes are the same as those released by TAIR9. For At3g02410 and At5g15860, the two ORF sequences corresponded to At3g02410.1 http://www.arabidopsis.org and At5g15860.1 [21], the longer variants of the two genes. For this reason, in this study, we only focused our interests on the longer splice variants for both At3g02410 and At5g15860. The amino acid sequences were derived from these ORFs and multiple amino acid sequence alignment of the three proteins was performed using Clustal W2 program http://www.ebi.ac.uk/Tools/es/cgi-bin/clustalw2. Results revealed that these three proteins were highly similar: ICME-LIKE1 shared 59% and 61% amino acid identity with ICME-LIKE2 and ICME, respectively, while ICME-LIKE2 and ICME had 76% identity (figure 1A). PROSITE software analysis http://www.expasy.ch/prosite showed that all proteins contained a highly conserved catalytic triad: a serine, an aspartate and a histidine (figure 1A, indicated by #) as well as substrate binding pocket domains, GGA and QSA (figure 1A, indicated by red boxes). Conserved domain search http://www.ncbi.nlm.nih.gov/Structure/cdd/wrpsb.cgi showed that all three proteins belong to the esterase lipase superfamily (figure 1B), while ICME-LIKE2 and ICME contain carboxylesterases type-B serine active site with perfect pattern: F-[GR]-G-x (4)-[LIVM]-x-[LIV]-x-G-x-S-[STAG]-G http://www.expasy.ch/prosite.

Using these three proteins as search query, similar proteins with more than 50% identity from plant kingdom were retrieved from NCBI through Blastp [23] for the purpose of evaluating the evolutionary relationship among these proteins. Phylogenetic tree were constructed using several tree building programs, including Neighbor Joining (NJ), Minimum Evolution (ME) and Maximum Parsimony (MP), which are available at the MEGA4 website http://www.megasoftware.net. Clustering analysis showed that different methods yielded similar clustering patterns. The results obtained with NJ method were presented (figure 1C) and results obtained with ME and MP methods can be found in additional files 1 and 2, respectively. Results showed that the ICME and two ICME-like proteins together with similar proteins, NP _001132117 from *Zea mays*, BAF18320 and BAF20445 from *Oryza sativa*, CAO42055 from *Vitis vinifera*, and ABD96862 from *Cleome spinose*, could be classified into one group with 100% bootstrap support. The group is divergent from two other groups, including the previously reported 20 Arabidopsis Carboxylesterases (AtCXE) [24] and those that function as a suppressor of AvrBst-elicited resistance in Arabidopsis [25]. The analysis demonstrated that the ICME genes in Arabidopsis belong to a small gene family.

### ICMEs of Arabidopsis are targeted to ER and Golgi apparatus

Trans-membrane domain was predicted using several online programs listed in Table 1.

**Table 1:** Programs used to predict trans-membrane domain of ICMEs of *Arabidopsis*

*Programs and their websites*	*Proteins*	*No. of transmembrane domains*
DAS http://www.sbc.su.se/~miklos/DAS/	ICME-LIKE1	6 (loosing cutoff 1.7 ); 2 (strict cutoff 2.2)
	ICME-LIKE2	4 (loosing cutoff 1.7 ); 4 (strict cutoff 2.2)
	ICME	4 (loosing cutoff 1.7 ); 2 (strict cutoff 2.2)
HMMTOP http://www.enzim.hu/hmmtop/html/submit.html	ICME-LIKE1	2 (N-terminus: OUT)
	ICME-LIKE2	4 (N-terminus: IN)
	ICME	4 (N-terminus: IN)
SOSUI http://bp.nuap.nagoya-u.ac.jp/sosui/sosui_submit.html	ICME-LIKE1	2
	ICME-LIKE2	2
	ICME	NO
TMpred http://www.ch.embnet.org/software/TMPRED_form.html	ICME-LIKE1	4 (Inside to outside helices);5 (Outside to inside helices)
	ICME-LIKE2	5 (Inside to outside helices);5 (Outside to inside helices)
	ICME	5 (Inside to outside helices);7 (Outside to inside helices)
TMHHM http://www.cbs.dtu.dk/services/TMHMM-2.0/	ICME-LIKE1	1 (N-terminus: IN)
	ICME-LIKE2	1 (N-terminus: IN)
	ICME	1 (N-terminus: IN)

**Figure 2:**
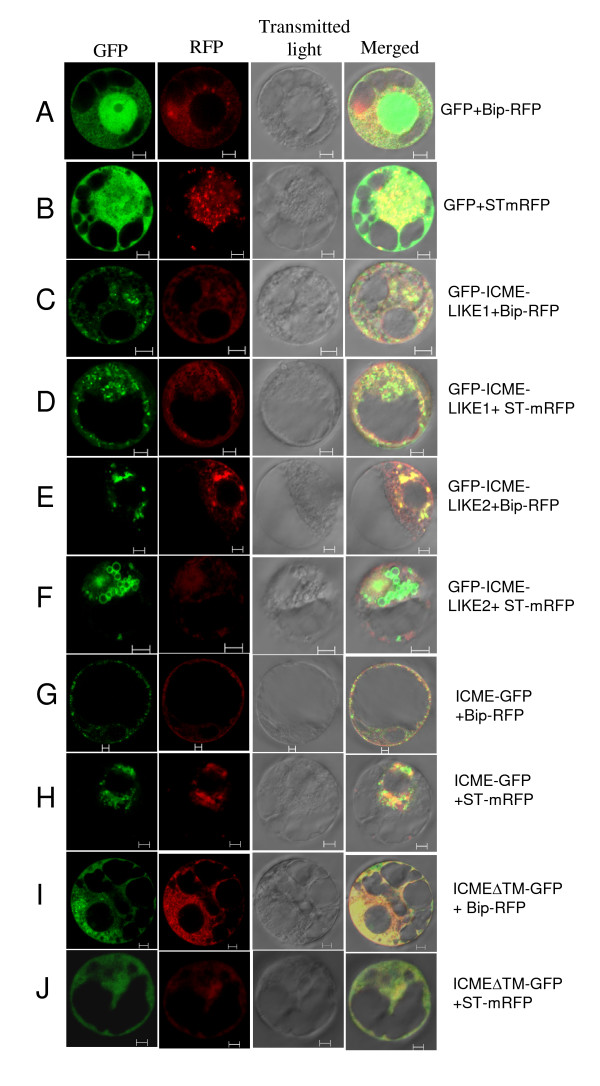
**Sub-cellular localization of GFP-tagged ICME and its homologs**. Protoplasts of *N. benthamiana* were co-transformed with free GFP, GFP-ICME-LIKE1, GFP -ICME-LIKE2, ICME-GFP, ICMEΔTM-GFP and ER marker BiP-RFP **(A, C, E, G and I) **or Golgi apparatus marker ST-mRFP **(B, D, F, H and J)**. For each combination shown to the right, images of GFP fluorescence, DesRed fluorescence, brightfield, and the merged were taken using a Bio-Rad confocal laser microscope MRC1024 and were displayed from left to right, respectively. Bar = 5 μm

As a result, except for SOSUI program that predicted ICME as a soluble protein, all other programs predicted that the three proteins all contain at least one trans-membrane domain (Table 1). To provide experimental evidence of the sub-cellular localization of ICME and its homologs, a transient expression of GFP-fused ICME and its homologs into onion epidermal cells and *N. benthamiana* protoplasts that isolated from suspension cultured cells, was performed. Similar sub-cellular localization patterns of the ICME family were obtained in these two systems (the protoplast system results are presented in figure 2 and the onion system results can be found in additional file 3). As shown in figures 2A and 2B, when co-expressing the free GFP gene with the ER or Golgi marker genes, the GFP fluorescence was spread throughout the cell (excluding vacuolar) with stronger signals in the nucleus (figures 2A and 2B, GFP channel), which was partially overlapped with the DesRed fluorescence emitted by the ER marker BiP-RFP (figure 2A, merged channel) and Golgi marker ST-mRFP (figure 2B, merged channel). When co-expressing the GFP fused ICME-LIKE1 with the ER and Golgi markers in protoplasts, besides small green punctates, the GFP fluorescence was predominantly restricted in the ER and Golgi apparatus, which was confirmed by co-localizing with the ER marker BiP-RFP (figure 2C, merged channel) and the Golgi marker ST-mRFP (figure 2D, merged channel). ICME-LIKE2 showed as an ER-resident protein (figure 2E) with partially localizing in the Golgi apparatus (figure 2F). Some fluorescent vesicles were observed surrounding the Golgi apparatus after GFP was fused to ICME-LIKE2 in some protoplasts (figure 2F, GFP channel). Similarly, ICME was predominantly localized in the ER and Golgi apparatus (figures 2G and 2H).

When the trans-membrane domain of the ICME family was predicted by using TMHHM program, both ICME and its homologs were found to contain one trans-membrane domain (Table 1). For ICME, amino acids 102 to 124 were predicted as a trans-membrane helix. When this region was deleted and the mutant was fused to GFP followed by expression in protoplasts, the GFP fluorescence was still limited in ER and Golgi apparatus, which was confirmed by co-localizing with the ER and Golgi apparatus makers (figures 2I and 2J).

### Tissue-specific expression patterns of ICME gene family

To examine the tissue-specific expression of *ICME *and its homologs, total RNA was extracted from different tissues including roots, rosette leaves, cauline leaves, stems, flowers, and siliques of 4-5 weeks old plants, followed by further treatment with DNase to remove the possible trace genomic DNA. One microgram of DNA-free RNA was converted into cDNA and semi-quantitative RT-PCR or real-time quantitative RT-PCR (qPCR) was performed.

**Figure 3:**
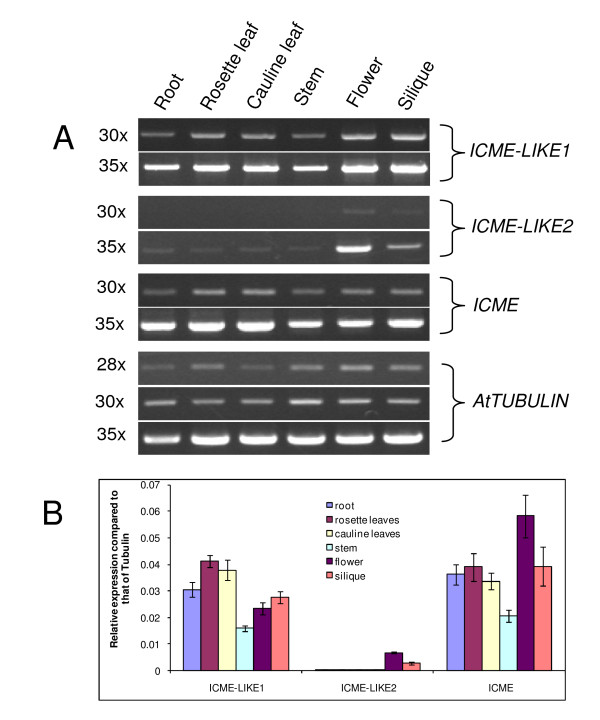
**Tissue-specific express patterns of ICME and its homologs**. Transcript level of *ICME *and its homologs were evaluated across different tissues including roots, stems, rosette leaves, cauline leaves, flowers, and siliques by semi-quantitative RT-PCR **(A) **and real-time quantitative RT-PCR **(B)**. For semi-quantitative RT-PCR, different PCR cycles were shown to the left; for real-time quantitative RT-PCR, the relative expression of *ICME *and its homologs compared to that of tubulin gene was shown as mean and SD (n = 3).

Both semi-quantitative RT-PCR (figure 3A) and qPCR (figure 3B) results showed that all ICME genes are constitutively expressed in all examined tissues with differentiated expression levels (figures 3A and 3B). The steady-state messenger RNA (mRNA) level of *ICME *was low in the stems but high in the reproductive organs including flowers and siliques (figures 3A and 3B). For *ICME-LIKE1*, it showed the highest mRNA level in the leaves but the lowest in the stems (figures 3A and 3B). Overall, transcript abundance of the *ICME-LIKE2 *gene was quite low; the mRNA level of *ICME-LIKE2 *was much lower than that of *ICME *and *ICME-LIKE1 *in all tested tissues (figures 3A and 3B), which was hardly detectable, especially in the vegetative organs including roots, leaves and stems, with semi-quantitative RT-PCR of less PCR cycles (figures 3A and 3B).

### Expression profiles of ICME gene family in response to abiotic stresses and ABA

**Figure 4:**
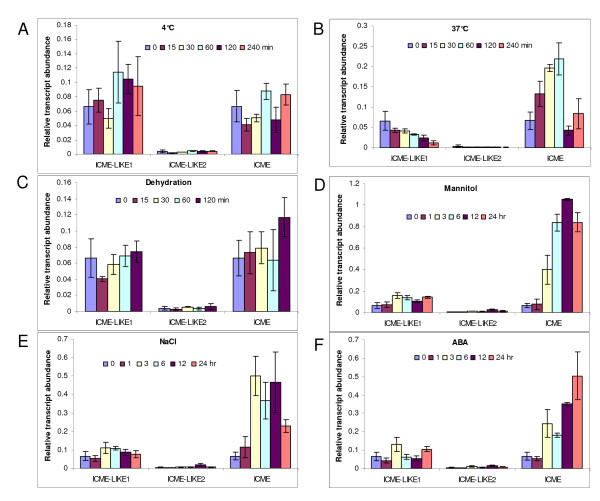
**Expression profiles of *ICME *and its homologs in response to abiotic stresses and ABA**. Seven-day-old seedlings were subjected to different abiotic stresses including cold at 4°C **(A)**, heat at 37°C **(B)**, drought **(C)**, 400 mM mannitol **(D)**, 200 mM NaCl **(E)**, and 50 μM ABA **(F) **for different time indicated. The transcripts were determined consequently by real-time quantitative RT-PCR, and the relative expression of *ICME *and its homologs compared to that of Actin8 gene was shown as mean and SD (n = 3).

Time-course expression profiles of the ICME gene family in response to different abiotic stresses including cold (4°C), heat (37°C), drought (dehydration), mannitol (400 mM), NaCl (200 mM), and ABA (50 μM) were determined using qPCR. Overall, cold treatment resulted in no significant expression change of the *ICME *family (figure 4A). The *ICME-LIKE1 *expression was slightly up-regulated by cold treatment after 60 min. In contrast, the expression of *ICME-LIKE2 *gene was slightly down-regulated by cold treatment after 15 min and recovered to the pre-treatment level after 60 min. Similar to the *ICME-LIKE2 *gene, under cold treatment, the *ICME *gene was slightly down-regulated quickly and then up-regulated.

In contrast to the response to the cold treatment, the expression of the *ICME-LIKE1 *gene was down-regulated during heat treatment (figure 4B), which could be applied to the *ICME-LIKE2 *gene (figure 4B). However, upon heat treatment, transcript of *ICME *showed a quick and transient increase. After 60 min of heat treatment, the *ICME *expression reached the maximum (figure 4B). At 120 min of heat treatment, the *ICME *expression was recovered to near the pre-treatment levels. From the expression profile, it is obvious that *ICME *is a quick and early response gene during heat stress, indicating that ICME may play an important role in the response of plants to heat stress.

When suffered from drought stress, both *ICME *and its homologs exhibited similar responses. Overall, the transcript abundance of these genes was not changed significantly except that at 15 min and 120 min of drought stress. The transcripts of *ICME-LIKE1 *and *ICME-LIKE2 *decreased at 15 min of treatment; whereas the transcripts of *ICME *and *ICME-LIKE2 *increased at 120 min after treatment (figure 4C).

As shown in figure 4D, in general, both *ICME *and its homologs were elevated by osmotic stress induced by mannitol treatment with differential levels of increase. Among them, the *ICME *gene showed the most pronounced up-regulation. At 12 hr of treatment, the expression of ICME reached the maximum with its transcript abundance being at least 10 times of that of the pre-treatment. At 3 hr of treatment, the expression of *ICME-LIKE1 *was maximally induced and this induction was lasted for 21 hrs. The *ICME-LIKE2 *gene was induced at 3 hr of treatment and was maximally induced at 12 hr of treatment.

Upon NaCl treatment, the expression profile of *ICME *and its homologs was similar to that in response to mannitol treatment. As shown in figure 4E, both *ICME *and its homologs were up-regulated by NaCl treatment, with *ICME *showing the strongest up-regulation. Moreover, the transcript of *ICME *increased quickly. The expression of *ICME *started increase at 1 hr of treatment and reached the maximum at 3 hr. Similarly, after 3 hr treatment, the expression of *ICME-LIKE1 *was maximally induced and this induction was lasted until 24 hr of treatment. In contrast, *ICME-LIKE2 *was induced at 3 hr of treatment and the expression reached the maximum at 12 hr of treatment.

As shown in figure 4F, the expression of both *ICME *and its homologs was increased in response to ABA. Among them, *ICME *and *ICME-LIKE2 *showed the most and the least pronounced up-regulation by ABA, respectively. Interestingly, transcript abundance of *ICME-LIKE2 *fluctuated during the course of ABA treatment, i.e, the transcript increased at 3 hr and backed to the pre-treatment level at 6 hr and then increased again at 24 hr after treatment. In contrast, the transcript of *ICME *showed a regular pattern; it increased at 3 hr and reached the maximum at 24 hr of ABA treatment.

### Identification of T-DNA insertion lines

**Figure 5:**
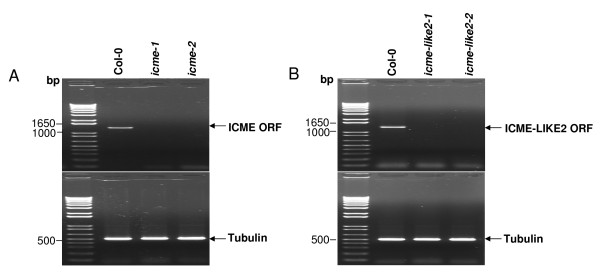
**Expressions of *ICME *(A) and *ICME-LIKE2 *(B) in wild type and mutants were analyzed by RT-PCR**. DNase-treated total RNA from flowers was converted to cDNA and the full-length ORF was amplified with 40 PCR cycles. Tubulin transcript abundance was used as an internal control.

To explore the biological functions of the ICME family, T-DNA insertion mutant lines were obtained from the Arabidopsis Biological Resource Center (ABRC, Ohio State University, Columbus). For each gene, two different T-DNA insertion lines were analyzed. A homozygous T-DNA insertion line, SALK_008773, with a T-DNA inserted at the 3' UTR of *ICME-LIKE1 *was identified by genomic DNA PCR and sequencing. However, this insertion neither disrupts the expression nor causes down-expression of *ICME-LIKE1*. Another line, SALK_011036 (putatively with a T-DNA inserted at the 5^th ^intron according to ABRC), was identified without any insertions at *ICME-LIKE1*, although the seeds could grow on Kanamycin containing MS medium and the transcript of Neomycin Phosphotransferase gene was also amplified by RT-PCR from total RNA extracted from this line. These results suggested that this line is a T-DNA insertion line with the T-DNA inserted at other unknown position. In contrast, the homozygous T-DNA insertion lines, SALK_043448 (called *icme-like2-1 *here) and SALK_002648 (called *icme-like2-2 *here) with a T-DNA inserted at the 8^th ^and 10^th ^intron of *ICME-LIKE2*, respectively, were identified by genomic DNA PCR and sequencing (additional file 4). The expression of *ICME-LIKE2 *was disrupted by both insertions (figure 5). Similarly, the homozygous T-DNA insertion lines, SALK_010304 (also called *icme-1 *[22]) and SALK_075701 (called *icme-2 *here) with a T-DNA inserted at the 1^st ^intron and 11^th ^exon of *ICME*, respectively, were identified by genomic DNA PCR and sequencing (additional file 5). Gene expression analysis showed that no transcripts (full-length ORF) of *ICME *were detected in the two homozygous mutants (figure 5).

Overall, *icme-like2-1, icme-like2-2, icme-1 and icme-2 *mutants are not distinguishable from wild-type plants in appearance under normal growth conditions (additional file 6).

Moreover, all of them exhibited similar responses when subjected to drought and salt stresses.

### Disruption of ICME-LIKE2 expression leads to increased sensitivity to ABA but slightly decreased to salt and osmotic stresses in seed germination

**Figure 6:**
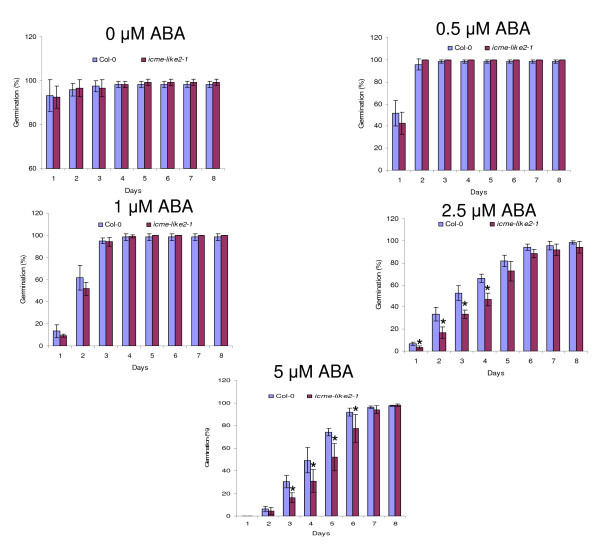
**The *icme-like2-1 *mutant showed increased sensitivity to ABA in seed germination**. Seeds of wild-type and *icme-like2-1 *mutant were sown on 0.5× MS medium with different concentration of cis ABA and stratified at 4°C for 3 days, then transferred to growth room under conditions of 16 hr light/8 hr dark cycle at 22°C. Seed germination was scored every day since transferring. All assays were repeated at least three times with similar results. More than 108 seeds were used in each experiment. Asterisks represent significant differences from the wild type of P < 0.05 as determined by Student's t test.

Seed germination of the wild type and *icme-like2 *mutants in response to ABA, salt and osmotic stresses was examined. Both *icme-like2-1 *and *icme-like2-2 *showed similar responses to the treatments. As shown in figure 6, in the absence of ABA, no differences of germination were observed between wild type and *icme-like2-1 *mutant. By contrast, disruption of *ICME-LIKE2 *expression leads to an increased sensitivity to ABA in seed germination, and the ABA-sensitive phenotype was apparent at all concentrations of ABA tested. On the other hand, the wild-type and the *ICME-LIKE2 *mutant showed no significant differences of cotyledon greening and seedling root growth (data not shown). In addition, in the low concentration of exogenous ABA, the difference was only observed in a short span during the early time of seed germination; whereas the differences were lasted for a much longer time in the high concentrations of ABA.

**Figure 7:**
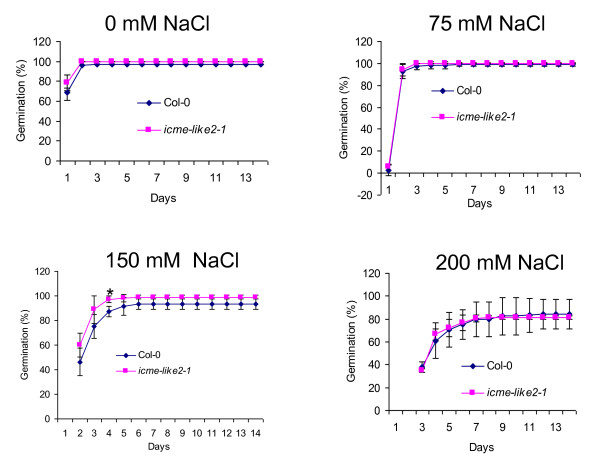
**The *icme-like2-1 *mutant showed slightly decreased sensitivity to salt stress in seed germination**. Seeds of wild-type and *icme-like2-1 *mutant were sown on MS medium with different concentration of NaCl and stratified at 4°C for 3 days, then transferred to growth room under conditions of 16 hr light/8 hr dark cycle at 22°C. Seed germination was scored every day since transferring. All assays were repeated at least three times with similar results. More than 108 seeds were used in each experiment. Asterisks represent significant differences from the wild type of P < 0.05 as determined by Student's t test.

**Figure 8:**
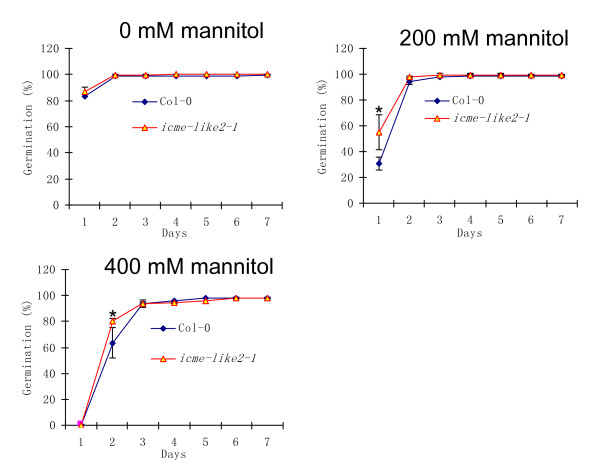
**The *icme-like2-1 *mutant showed slightly decreased sensitivity to osmotic stress in seed germination**. Seeds of wild-type and *icme-like2-1 *mutant were sown on MS medium with different concentration of mannitol and stratified at 4°C for 3 days, then transferred to growth room under conditions of 16 hr light/8 hr dark cycle at 22°C. Seed germination was scored every day since transferring. All assays were repeated at least three times with similar results. More than 108 seeds were used in each experiment. Asterisks represent significant differences from the wild type of P < 0.05 as determined by Student's t test.

In contrast to respond to ABA, overall, the *icme-like2-1 *mutant showed slightly decreased sensitivity to salt (figure 7) and osmotic (figure 8) stresses in seed germination compared to the wild type. But these differences are significant only under specific concentration and stage (labeled as * in figures 7 and 8), suggesting that ICME-LIKE2 exerts its role in specific conditions and developmental stage in response to environmental stimuli. Similarly, the wild-type and the *ICME-LIKE2 *mutants showed no significant differences of cotyledon greening and seedling root growth in response to these stresses.

## Discussion

ICME is involved in the process of demethylating the prenylated proteins in eukaryotic cells. An ICME was recently reported in Arabidopsis and its related gene was isolated [21]. Membranes isolated from *S. cerevisiae *cells expressing the *ICME *gene contain specific enzyme activity for relevant prenylcysteine methylesters [21]. Overexpression of ICME resulted in an ABA hypersensitive phenotype in stomatal closure and seed germination [22]. Moreover, this gene was up-regulated by ABA in Arabidopsis seedlings [22]. These results indicate that the demethylation of prenylated proteins is involved in at least some aspects, if not all, of ABA responses and ICME is a positive regulator of ABA signaling.

Structurally, both ICME and its homologs in this study belong to the esterase lipase superfamily containing two highly conserved regions: (1) the GXSXG motif containing the Ser nucleophile (figure 1A); and (2) the conserved catalytic triad possessing the nucleophile-acid-His-ordered sequence (Ser, Asp, and His) (figure 1A). Initially, we identified ICME as an acetylcholinesterase (AChE)-like protein with the attempt to clone the gene encoding AChE-like protein in *Planta *since the AChE-like activity was detected in maize seedlings [26]. Until now, it is still unclear whether ICME and its two homologs, ICME-LIKE1 and ICME-LIKE2, possess enzyme activity to hydrolyze acetylthiocholine. In 2005, an AChE (NP_001105800) with 394 amino acids in length from maize seedlings was identified and the related gene was cloned [27]. This AChE contains the same conserved catalytic core as in the animal AChE, possessing the enzyme activity of hydrolyzing acetylthiocholine and propyonylthiocholine, but not S-butyrylthiocholine. Moreover, its enzyme activity was totally inhibited by neostigmine bromide, a specific inhibitor of animal AChE [27]. However, the amino acid sequence of this AChE showed no apparent similarity with that of the animal enzyme. Based on these, the authors proposed that this AChE family is a novel family of enzymes in plant kingdom. Amino acid sequence alignment showed ICME and its homologs were exhibited very lower similarity with that of the maize AChE (Data not shown), suggesting that ICME and its homologs are not AChE-like proteins. In 2003, twenty carboxylesterases in Arabidopsis (AtCXE) were systemically mined from Arabidopsis genome, and the expression profile across a range of tissues was detected by RT-PCR [24]. One of AtCXEs, AtCXE12, was reported to possess enzyme activity for hydrolyzing the pro-herbicide methyl-2,4-dichlorophenoxyacetate to the phytotoxic acid 2,4-dichlorophenoxyacetic acid [28]. It remains unknown whether ICME and its homologs have such enzyme activity to hydrolyze pro-herbicide methyl-2,4-dichlorophenoxyacetate. Although all the 20 AtCXEs contains the conserved catalytic triad, amino acid sequence alignment showed that ICME and its homologs are divergent from those AtCXEs. Moreover, none of AtCXEs is larger than 400 amino acids in length but ICME and its homologs are larger than 400 amino acids in length. In addition, ICME and its homologs are lack of the conserved sequence surrounding the motif known as the oxyanion hole in AtCXEs. Phylogenetic analysis further showed that ICME and its homologs and AtCXEs are clustered into different groups, respectively (figure 1C). These indicate that ICME and its homologs may not possess the enzyme activity of AtCXEs. In 2007, another carboxylesterase, SOBER1 (At4g22300), was identified as suppressors of AvrBsT-elicited resistance in Arabidopsis [25]. SOBER1 and its homologs are also clustered into an independent group (figure 1C). Enzymes in this group are smaller than that of AtCXEs and ICME and its homologs are less than 300 amino acids in length. Besides the conserved catalytic triad, enzymes in this group contain another unique "LHGLGD" motif in the N-terminal region that is not present in the ICME and its homologs. In summary, bioinformatic analysis in this study and previous biochemical evidence [21] suggest that ICME and its homologs may comprise a novel small subfamily of carboxylesterase in plant kingdom, possibly with the prenylated protein as its specific substrate. Given the high degree of similarity among ICME, ICME-LIKE1, and ICME-LIKE2 in terms of sequence and subcellular localization (figure 2 and additional file 3), it is reasonable to predict that ICME-LIKE1 and ICME-LIKE2 possess ICME activity. Direct biochemical evidence of ICME activity of the ICME-like proteins is necessary for better understanding their functions in plants in the future.

Documented data suggested that protein prenylation and subsequent mature processing steps promote its attachment to membrane and, in some cases, protein-protein interaction by increasing its C-terminal hydrophobicity [1-3]. In other words, with high possibility, a maturely prenylated protein is a membrane protein or is associated with the membrane system. ICME is involved in demthylation of the prenylated proteins, the only reversible process during protein prenylation modification. These suggest that ICME is likely a membrane-resident protein. Previous biochemical analysis also showed that enzyme activity of ICME was detected in membrane fragment [20,21]. In the current study, more precise sub-cellular localizations of ICME family were provided using GFP tagged proteins. Initially, we fused all ICME and its homologs in frame to the N-terminus of GFP. Except for ICME, we failed to detect any fluorescent signal for GFP tagged ICME-LIKE1 and ICME-LIKE2, suggesting that N-terminus fused GFP may be unstable. We then fused these two proteins to the C-terminus of GFP and successfully detected the fluorescence. The fluorescent signals clearly revealed that proteins of the ICME family are intramembrane proteins predominantly localizing at the ER and Golgi apparatus, which was confirmed by co-expression of ER and Golgi markers, BiP-RFP and ST-mRFP, respectively (figure 2 and additional file 3). These results also suggest that many prenylated proteins, at least those that being in the processes of demthylation, are targeted to ER and Golgi apparatus. Subcellular proteomics of ER and Golgi apparatus may provide more information about protein prenylation and its mature process, which will be helpful to explore the biological functions of protein prenylation.

When transiently expressing GFP-ICME-LIKE2, some green vesicles surrounding the Golgi apparatus were observed in some protoplasts (figure 2). At present, the significance of these vesicles is unclear. We speculated that (1) these vesicles were secreted from ER and being sorted to the Golgi apparatus for its post-translational modifications. Motif scanning showed that ICME-LIKE2 could be modified by glycosylation (at the 47^th ^and 213^th ^amino acids Asn) and other potential manners http://myhits.isb-sib.ch/cgi-bin/motif_scan; (2) these vesicles modified in the Golgi apparatus were being sorted to its final target, ER. Further cell biology experiments, especially pharmacogenetic analysis, are needed to reveal the mechanisms underlying these vesicles.

Contrary results were obtained in predicting the trans-membrane domain of ICME by using different programs (Table 2). Zero and five transmembrane domains were predicted for ICME with SOSUI and TMpred programs, respectively. Deleting the putative transmembrane helix, amino acids 102^nd ^to 124^th^, did not block its ER-targeting. It can be concluded from this that ICME is an intramembrane protein containing at least two transmembrane domains and predominantly localizing in the ER and Golgi apparatus.

**Table 2:** Primers used in this study

*Name*	*Sequence (5' to 3')*	*Use*
1gRTF	GCACCATTGTAGAGCAGGTCA	Semi-quantitative
1gRTR	ACCAATCGCTTCTTGATCGTC	RT- PCR
3gRTF	TACATACCGCCAACGAGTGAT	Semi-quantitative
3gRTR	GCTACAAGAACCGTATCGAAG	RT- PCR
5gRTF	TCAAAGCAAGTCCGGAGGAGT	Semi-quantitative
5gRTR	TTTGGTCAGCCCATCATTGTC	RT- PCR
TuRTF	AGAACACTGTTGTAAGGCTCAAC	Semi-quantitative
TuRTR	GAGCTTTACTGCCTCGAACATGG	RT- PCR
5gNcoI F	CGGCCATGGAAATGCATTCGCCTCTTCAGACTCA	pICME-GFP construction
5gNcoI R	TTGCCATGGCGAAAGGGCTAATCTCACGAG	
1gBglII F	GCCAGATCTATGCCGTCGCAGATTCTCCAAAT	pGFP-ICME-LIKE1 construction
1gSpeI R	GTGACTAGTTAGAACGGACTGACCCAGTGAGCCAACTT	
3gBglII F	GTCTAGATCTATGCAGTTGTCTCCGGAACG	pGFP-ICME-LIKE2 construction
3gSpeI R	TTAACTAGTCAAAAAGGGCTGACCCTGCCAGCCAGTT	
5gΔTM F1	GAAATGCATTCGCCTCTTCAG	pICMEΔTM-GFP construction
5gΔTM R1	CGGACTTGCTTTGAGAAAAATTTTGTCATCCATCGATAGC	
5gΔTM F2	GCTATCGATGGATGACAAAATTTTTCTCAAAGCAAGTCCG	
5gΔTM R2	AAGGCATGCTACAATGATATC	
1gqRTF	CTCACATCATCTTCCACCTAAATC	Real-time quantitative
1gqRTR	AAATCGAGAGAGAAGGGTCGT	PCR
3gqRTF	CCGATGTCTGAAAACAGAGAGG	Real-time quantitative
3gqRTR	CCGGTGAAGATAATCTGTTGG	PCR
5gqRTF	AGGATCCCTTACGAGGAGGT	Real-time quantitative
5gqRTR	AGCCAACGAGTCTTTGGTCAG	PCR
TuqRTF	GTGCTGAAGGTGGAGACGAT	Real-time quantitative
TuqRTR	AACACGAAGACCGAACGAAT	PCR
Actin8qRTF	TTACCCGACGGACAAGTGATC	Real-time quantitative
Actin8qRTR	ATGATGGCTGGAAAAGGACTTC	PCR
P2	tcacttccataatcggggtctg	T-DNA screening
LBa1	TggTTCACgTAgTgggCCATCg	T-DNA screening

So far, the molecular information about the *ICME *family is unavailable except that the expression of *ICME *was up-regulated in Arabidopsis seedlings by ABA after 24 hrs. There is also a lack of information about the expression profiles for *ICME-LIKE1 *and *ICME-LIKE2 *in the public database. Study here showed that the members of the *ICME *family are expressed across a range of tissues with differential expression level (figure 3), indicating that ICME and its homologs may have broad functions in Arabidopsis. On the other hand, this also suggests the existence of functional redundancy among the ICME family. The base transcript of *ICME-LIKE2 *is quite low, indicating it is a low abundance gene. The low detection sensitive of northern blotting may explain why there is no public microarray data available. It is also possible that the expression of *ICME-LIKE2 *is indistinguishable from *ICME *due to their high similarity, leading to lack of expression information on *ICME-LIKE2 *in the public microarray data. Lacking of the expression information on *ICME-LIKE1 *in the public microarray data is unexpected since its base transcript abundance is similar to that of *ICME *(figure 3). Moreover, the transcript increased in response to cold, salt, osmotic stresses and ABA treatment (figure4). The most possible explanation for this is that the expression of *ICME-LIKE1 *is indistinguishable from *ICME *because of their high similarity.

By time-course assays, study here not only confirmed the previous data that *ICME *was up-regulated by ABA treatment after 24 hr [22], but also provided that *ICME *is also a stress-regulated gene, which is consistent with the public micoarray data https://www.genevestigator.com/gv/directlink.jsp?geneIDs=AT5G15860. In addition, *ICME* was dramatically up-regulated by other abiotic stresses, including heat, mannitol, and salt treatment, suggesting that ICME might be involved in stress response. Indeed, prenylated proteins have been implicated in the processes in response to heat stress [29]. Although study here showed that disruption the expression of ICME (figure 5) caused no different phenotypes between wild type and mutant plants under normal and drought and salt stress conditions (additional file 6 and data not shown), whether the ICME activity is required for tolerating heat stress is to be confirmed with *icme *mutants, *icme *and *icme-like2 *double mutants and transgenic plants bearing over-expression of ICME.

By now, the biological functions of the ICME family are largely unclear; the only confirmed function is that the over-expression of ICME caused an ABA hypersensitive phenotype in stomatal closure and seed germination while *icme-1 *mutant exhibited an ABA insensitive phenotype [22]. This is different from our data using ICME-LIKE2 mutants. Knocking out the expression of ICME-LIKE2 resulted in increased sensitivity to ABA in seed germination (figure 6). These suggest that ICME and ICME-LIKE2 may have different roles in seed germination in response to ABA. However, except that the disruption of *ICME-LIKE2 *expression leads to increased sensitivity to ABA and slightly decreased to salt and osmotic stresses in seed germination, no significant differences were observed between wild type and mutant plants in terms of other physiological functions tested. Similarly, no significant differences were observed between wild type and ICME mutants in other biological process in previous study [22], which has been confirmed in the current study (additional file 6). This may be due to the existence of functional redundancy among the ICME family. Firstly, the expression of the ICME family is universal; secondly, the sub-cellular localizations of the ICME family are similar; thirdly, the expression patterns of the ICME family in response to abiotic stresses and ABA is similar; and finally, the members of the ICME family share high identity in amino acid and contain the conserved functional domain. The biological process of ICME-LIKE2 is worth further detailed study. Some important facts about ICME-LIKE2 include (1) it is a low abundance gene (figures 3 and 4); (2) its transcript abundance is induced by abiotic stresses and ABA (figure 4); (3) it forms some vesicles around ER (figure 2); and (4) null mutants of ICME-LIKE2 are available (figure 5). Based on these, it can be predicted that ICME-LIKE1 and ICME-LIKE2 are involved in ABA signaling and may also be involved in other stress responses. To better understand the biological functions of the ICME family, future works will be to identify null or knock-down mutant of *icme-like1 *and construct double and triple mutants of the ICME family.

## Conclusions

In this paper, the extended characterizations, sub-cellular localizations, expression profiles across different tissues and in response to abiotic stress and ABA of ICME and its homologs as well as their physiological functions are reported. These studies confirmed that there is a small ICME family with specific structural characterizations in Arabidopsis. All members of the family have similar sub-cellular localizations predominantly localizing at the ER and Golgi apparatus. The genes of the *ICME *family are expressed across a broad range of tissues. Upon stress stimuli and ABA treatment, most of the expressions of the *IMCE *family are up-regulated. Single mutants of icme and icme-like2 do not show significant effects in plant growth, development and responses to environmental stimuli. However, disruption of the *ICME-LIKE2 *expression leads to increased sensitivity to ABA and slightly decreased sensitivity to salt and osmotic stresses in seed germination. Double and triple mutants of the *ICME *family may be better option to explore their functions in future. The ABA and abiotic stress regulated genes reported here are potentially useful resources for transgenic crops against drought and other stresses.

## Methods

### Plant growth and treatments

Arabidopsis thaliana ecotype Columbia was used throughout the current study.

Mutant seeds *icme-like1-1 *(SALK_008773), *icme-like1-2 *(SALK_011036), *icme-like2-1 *(SALK_043448), *icme-like2-2 *(SALK_002648), *icme-1 *(SALK_010304), and *icme -2 *(SALK_075701) were obtained from the Arabidopsis Biological Resource Center (ABRC, Ohio State University, Columbus). Seeds were surface-sterilized and sown on solid agar plates containing 1× Murashige and Skoog (MS) salts, 1% (w/v) sucrose, 0.8% agar adjusted to pH5.7. After stratified at 4°C for 3 days, they were transferred to a growth chamber at 22°C under a 16-h light/8-h dark photoperiod at 130 μEm^-2^sec^-1^.

Seven-day-old seedlings were used for different treatments: for salt, osmotic, and ABA treatment, seedlings sowed on filters (which were put on 1× MS agar media) were transferred to MS liquid media containing 200 mM NaCl, 400 mM mannitol, or 50 μM ABA for different time indicated. For cold and heat treatments, the plates were transferred to 4°C freezer or 37°C growth chamber. For dehydration test, seedlings were transferred to Petri dish covers and left at growth chamber for different length of times. After treatment, seedlings were collected in liquid nitrogen and stored at -80°C for RNA extraction.

### Verification of the T-DNA insertion mutants

Homozygous mutant was identified by PCR from genomic DNA using gene-specific primers (Table 2) and T-DNA left border primers LBa1, and analyzed further by DNA sequencing to confirm the insertion of the T-DNA in the gene.

### Plasmid construction

ORFs encoding the full-length ICME-LIKE1, ICME-LIKE2, and ICME were amplified by PCR with the specific primer pairs listed in Table 1. The PCR products were inserted in-frame to the C-terminus (for ICME-LIKE1 and ICME-LIKE2) or N-terminus (for ICME) of the GFP gene in plasmid pAVA121 [30]. This plasmid encodes an enhanced free GFP under the guide of 35 S promoter of *Cauliflower mosaic virus *and the NOS terminator, designated as pGFP here. The PCR products of ICME-LIKE1 and ICME-LIKE2 amplified with primer pairs 1gBglII F/1gSpeI R and 3gBglII F/3gSpeI R, respectively, were digested with BglII and SpeI and ligated to pGFP digested with BglII and XbaI to create pGFP-ICME-LIKE1 and pGFP-ICME-LIKE2, respectively. Similarly, PCR product of ICME from primer pair 5gNcoI F/5gNcoI R was digested with NcoI and inserted to pGFP, which was predigested with NcoI and followed by further dephosphorylation with shrimp alkaline phosphatase (Promega, USA). Constructs with correct orientation were selected by restriction assays and designated as pICME-GFP.

Based on pICME-GFP, the putative transmembrane domain (TM), amino acids 102 to 124, was deleted to create plasmid pICMEΔTM-GFP by overlapping PCR. Briefly, the first fragment was generated with primer pair 5gΔTMF1/5gΔTMR1; while the second fragment was synthesized with primer pair 5gΔTMF2/5gΔTMR2 (primer 5gΔTMF2 is complementary exactly to the primer 5gΔTMR1 in nucleotide sequence).

Next, overlapping PCR was performed with primer pair 5gΔTMF1/5gΔTMR2 using the mixture of the first and the second fragments as the PCR template. Following deletion, the final PCR product was digested with NsiI and NheI and subcloned into pICME-GFP digested with the same set of restriction enzymes to replace the corresponding fragment of wild type cDNA. All the final constructs were verified by DNA sequencing.

### Protoplast isolation, transformation, and Confocal Microscopy

Protoplasts of N. benthamiana were isolated from suspension cell lines and co-transformed with plasmids bearing free GFP or GFP tagged ICME or its homologs and plasmids BiP-RFP or ST-mRFP [31], which are widely used as the ER and Golgi apparatus markers, respectively) as described earlier [32]. Confocal microscope images were taken using a confocal laser microscope MRC1024 under a ×63 water objective. DesRed images were captured in the 560- to 615-nm range after excitation at 543 nm with a HeNe laser beam. The GFP images were captured in the 505- to 530-nm range after excitation at 488 nm with an argon laser beam.

### Particle bombardment

Onion epidermal cells were co-bombarded with 2.5 μg of plasmids bearing free GFP, GFP tagged ICME or its homologs and plasmids BiP-RFP or ST-mRFP [30]. All the plasmids were coated onto 1-μm gold particles and delivered into onion epidermal cells at a pressure of 900 psi by PDS 1000/He particle delivery system (BioRad, U.S.A). After bombardment, onion slices of 2 × 2 cm were placed on a plate containing 1× MS salts, 30 g/L of sucrose, 1.5% agar, pH = 5.7. After at least 24 hr, the epidermis was removed from the onion slice and observed using a Confocal Laser Scanning Microscope.

### Total RNA isolation, Semi-Quantitative RT-PCR and Real-Time Quantitative RT-PCR

Total RNA was isolated from a broad range of tissues of 4-5 weeks old plants and seedlings of various treatments using RNeasy Mini Kit (QIAGEN) and further treated with DNase using the TURBO DNA-free Kit (Ambion) according to the manufacturer's manual. About 800 ng DNA-free total RNA was converted to cDNA using Oligo-dT (20) primer and Superscript II reverse transcriptase (Invitrogen) according to the manufacturer. Briefly, after incubation at 50°C for 1 h followed by 85°C for 5 min, 1 μl of RNase H was added and incubated for 20 min at 37°C. The cDNA was used as a PCR template for both Semi-Quantitative RT-PCR and Real-Time Quantitative RT-PCR.

For Semi-Quantitative RT-PCR, β-tubulin was used as an internal control. Different PCR cycles (28, 30, and 35) were used for β-tubulin amplification to calculate the cDNA amount for gene-specific amplification. A total of 30 or 35 PCR cycles were used for ICMEs amplification. The PCR program used was as follows: pre-soaking in 94°C for 5 min, followed by different cycles of 94°C for 30 sec, 58°C for 30 sec and 72°C for 45 sec. A final incubation at 72°C for 7 min was included to complete product synthesis. PCR products were separated on a 1% agarose gel stained with ethidium bromide.

For Real-Time Quantitative RT-PCR, PCR amplification was performed in a 20 μl reaction system using SYBR Green PCR Master Mix (Applied Biosystems) with programs recommended by the manufacturer (2 min at 50°C, 10 min at 95°C and 40 cycles of 95°C for 15 sec and 60°C for 1 min). Three independent replicates were performed for each sample. The comparative CT method was used to determine the relative amount of gene expression, with the expression of tubulin (for tissue expression patterns) or *Actin8 *gene (for expression profiles responding to biotic stimuli) used as an internal control. The PCR was carried out in an ABI StepOne Real-Time PCR System (Applied Biosystems). All primers used in this study are listed in Table 2.

### Seed germination and early seedling development assays

#### Seed Germination

Plants of all genotypes were grown in the same conditions, and seeds were collected at the same time. For each comparison, seeds were planted on the same plate containing MS medium (0.5× MS salts, 1% Suc, and 0.8% agar) without or with different concentrations of ABA, NaCl or mannitol as indicated. Plates were stratified at 4°C in the dark for 3 d and transferred to 22°C with a 16-h-light/8-h-dark cycle. The percentage of seed germination was scored at the indicated times. Germination was defined as an obvious emergence of the radicle through the seed coat.

#### Cotyledon greening

To study the effect of ABA and other osmotic agents on cotyledon greening, seeds were sown on MS medium as described above. Three days after stratification, germinated seeds were transferred to medium containing different concentrations of ABA or other osmotic agents (NaCl and mannitol) as indicated. The percentage of cotyledon greening was recorded at 7 d after the end of stratification. Cotyledon greening is defined as obvious cotyledon expansion and turning green.

#### Seedling root growth

To study the effect of ABA and or other osmotic agents on seedling root growth, seeds were sown on MS plates vertically in growth chamber. Five-day-old seedlings were transferred to medium containing different concentrations of ABA or other osmotic agents for another 5 days and root growth was measured after the transfer.

### Drought, salt treatment and determination of transpiration rate

Drought tolerance test was examined in soil, 7-day-old seedlings were transplanted from MS plates to the soil for another 2 weeks under normal growth conditions. Plants were then subjected to progressive drought test by withholding water for specified times or rosette leaves of similar developmental stages (third to fifth true rosette leaves) were detached and placed abaxial side up on open Petri dishes and weighed at different time intervals at room temperature to determine transpiration rate. For salt treatment, 3-week-old plants were watered with salt solution with increased concentration from 50 mM to 200 mM every four days. To minimize experimental variations, the same numbers of plants were grown on the same tray. The entire test was repeated for a minimum of three times.

## Authors' contributions

PL performed bioinformatics analysis, sub-cellular localization, germination experiments, identified T-DNA mutants (ICME-LIKE1 and ICME-LIKE2 genes) and drafted the manuscript. WL conducted real-time PCR, identified T-DNA mutants (ICME gene) and participated in drafting the manuscript. HW performed the Phylogenetic tree construction with Minimum Evolution and Maximum Parsimony methods and aided in data analysis. WM conceived the study, assisted in designing the experiment, interpretation of the data, and finalizing the manuscript. All authors approved of the manuscript.

## Supplementary Material

Additional file 1 **Phylogenetic tree was constructed by Minimum Evolution method using MEGA4 program**. Phylogenetic relationships of ICME, ICME-like proteins and their homologs from *Zea mays, Oryza sativa, Vitis vinifera*, and *Cleome spinosa *as well as carboxylesterases from *Arabidopsis*. Click here for file

Additional file 2 **Phylogenetic tree was constructed by Maximum Parsimony method using MEGA4 program**. Phylogenetic relationships of ICME, ICME-like proteins and their homologs from *Zea mays, Oryza sativa, Vitis vinifera*, and *Cleome spinosa *as well as carboxylesterases from *Arabidopsis*. Click here for file

Additional file 3 **Sub-cellular localization of GFP-tagged ICME and its homologs**. Onion epidermal cells were co-transformed with free GFP, GFP-ICME-LIKE1, GFP -ICME-LIKE2, ICME-GFP and ER marker BiP-RFP (A, C and E) or Golgi apparatus marker ST-mRFP (B, D and F), respectively. For each combination shown on the right, images of GFP fluorescence, DesRed fluorescence, brightfield, and the merged were taken using a Zeiss confocal laser microscope LSM510 and were displayed from left to right, respectively. Click here for file

Additional file 4 **Identification of T-DNA insertion mutant lines of *ICME-LIKE2 *gene**. (A) Gene structure of the *ICME *(thick lines indicate exon and thin lines indicate intron) and the locations of the T-DNA insertion in the icme-1(SALK_043448) and icme-2 (SALK_002648) lines are shown. Primers used to screen are shown as arrows. (B) Homozygous T-DNA insertion lines were identified by Genomic-PCR. *icme-like2-1 *was identified by two primer pairs: (1) LBa1 and gene-specific forward primer 3gRTF for confirming T-DNA insertion and (2) 3gRTIF and 3gRTR for confirming homozygous T-DNA insertion. *icme-like2-2 *was identified by two primer pairs: (1) LBa1 and gene-specific reverse primer 3gRTR for confirming T-DNA insertion and (2) 3gRTF and 3gRTR for confirming homozygous T-DNA insertion. (C) The flanking sequence of the T-DNA insertion lines. The position of the T-DNA insertion was confirmed by DNA sequence analysis of the resultant PCR products. Sequences underlined referred to the left border of LB, while the italic ones referred to the genomic sequences. The bold ones indicated the additional sequences due to T-DNA insertion. Click here for file

Additional file 5 **Identification of T-DNA insertion mutant lines of *ICME *gene**. (A) Gene structure of the *ICME *(thick lines indicate exon and thin lines indicate intron) and the locations of the T-DNA insertion in the icme-1(SALK_010304) and icme-2 (SALK_075701) lines are shown. Primers used to screen are shown as arrows. **(B) **Homozygous T-DNA insertion lines were identified by Genomic-PCR. *icme-1 *was identified by two primer pairs: (1) LBa1 and gene-specific forward primer 5gNcoIF for confirming T-DNA insertion and (2) 5gNcoIF and 5gRTR for confirming homozygous T-DNA insertion. *icme-2 *was identified by two primer pairs: (1) LBa1 and gene-specific forward primer P2 for confirming T-DNA insertion and (2) P2 and 5gRTR for confirming homozygous T-DNA insertion. **(C) **The flanking sequence of the T-DNA insertion lines. The position of the T-DNA insertion was confirmed by DNA sequence analysis of the resultant PCR products. Sequences underlined referred to the left border of LB, while the italic ones referred to the genomic sequence. The bold ones indicated the additional sequences due to T-DNA insertion. Click here for file

Additional file 6 **Phenotypes, drought treatment and transpiration rates of wild type and mutants**. The up panels of (**A) **and (**B) **were 21-day-old plants under normal growth condition of different genotypes. The down panels of (**A**) and (**B) **were drought treated plants. For drought treatment, 21-day-old plants under normal growth condition of different genotypes were withheld water for 14 days. **(C) **Transpiration rates. Rosette leaves of the same developmental stages were excised from 21-old-day plants and weighed at various time points after detachment. Each data point represents the mean of duplicate measurements. Error bars represent SD (*n *= 3 each). Click here for file
