# Supplementary figures and images for: Characterization, sub-cellular localization and expression profiling of the isoprenylcysteine methylesterase gene family in Arabidopsis thaliana
Source: BMC Plant Biol. 2010 Sep 27;10:212. doi: 10.1186/1471-2229-10-212 (PMC3017835; doi:10.1186/1471-2229-10-212)

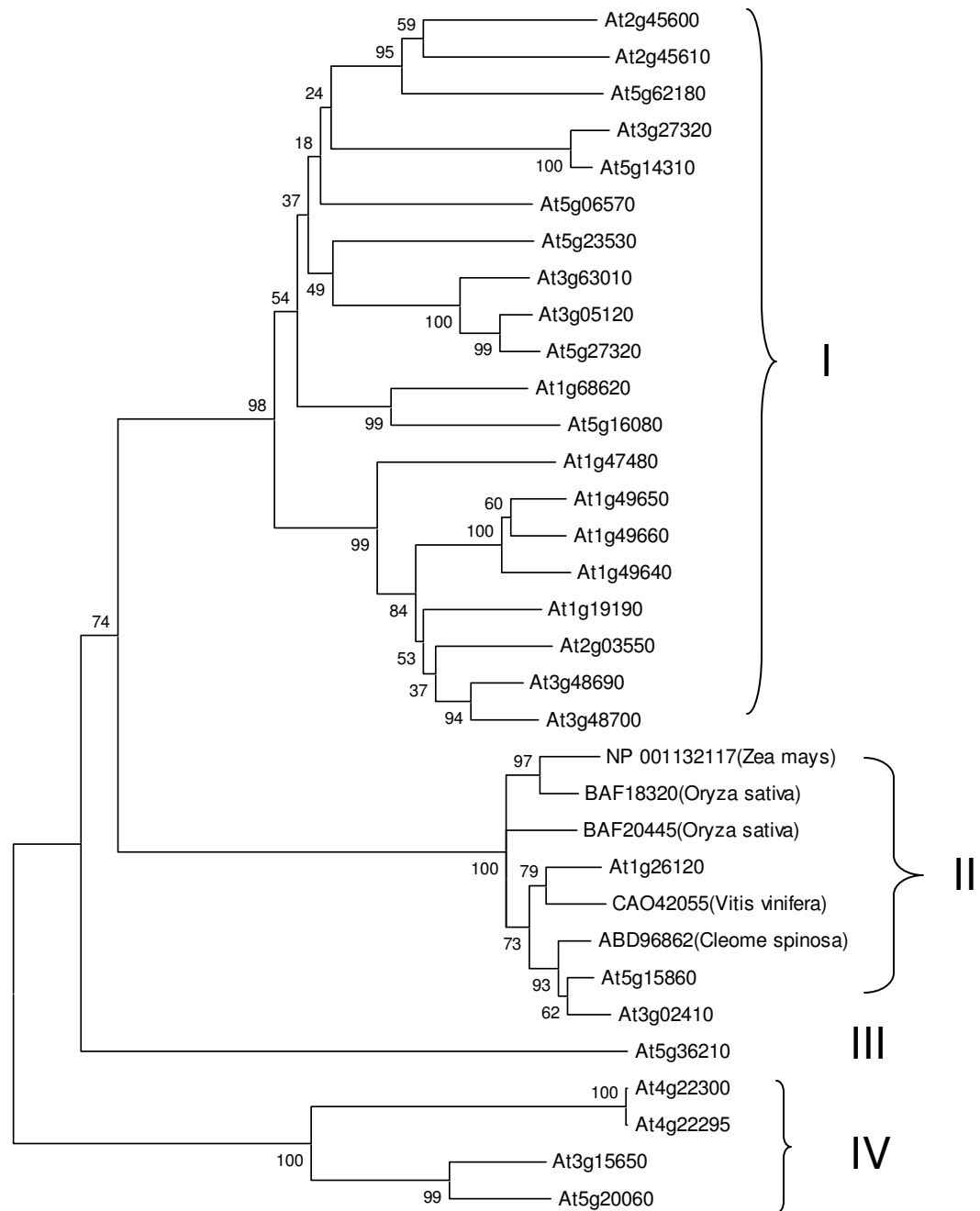

Supplement: Additional file 1 — Phylogenetic tree was constructed by Minimum Evolution method using MEGA4 program. Phylogenetic relationships of ICME, ICME-like proteins and their homologs from Zea mays, Oryza sativa, Vitis vinifera, and Cleome spinosa as well as carboxylesterases from Arabidopsis. [file 1471-2229-10-212-S1.PDF]

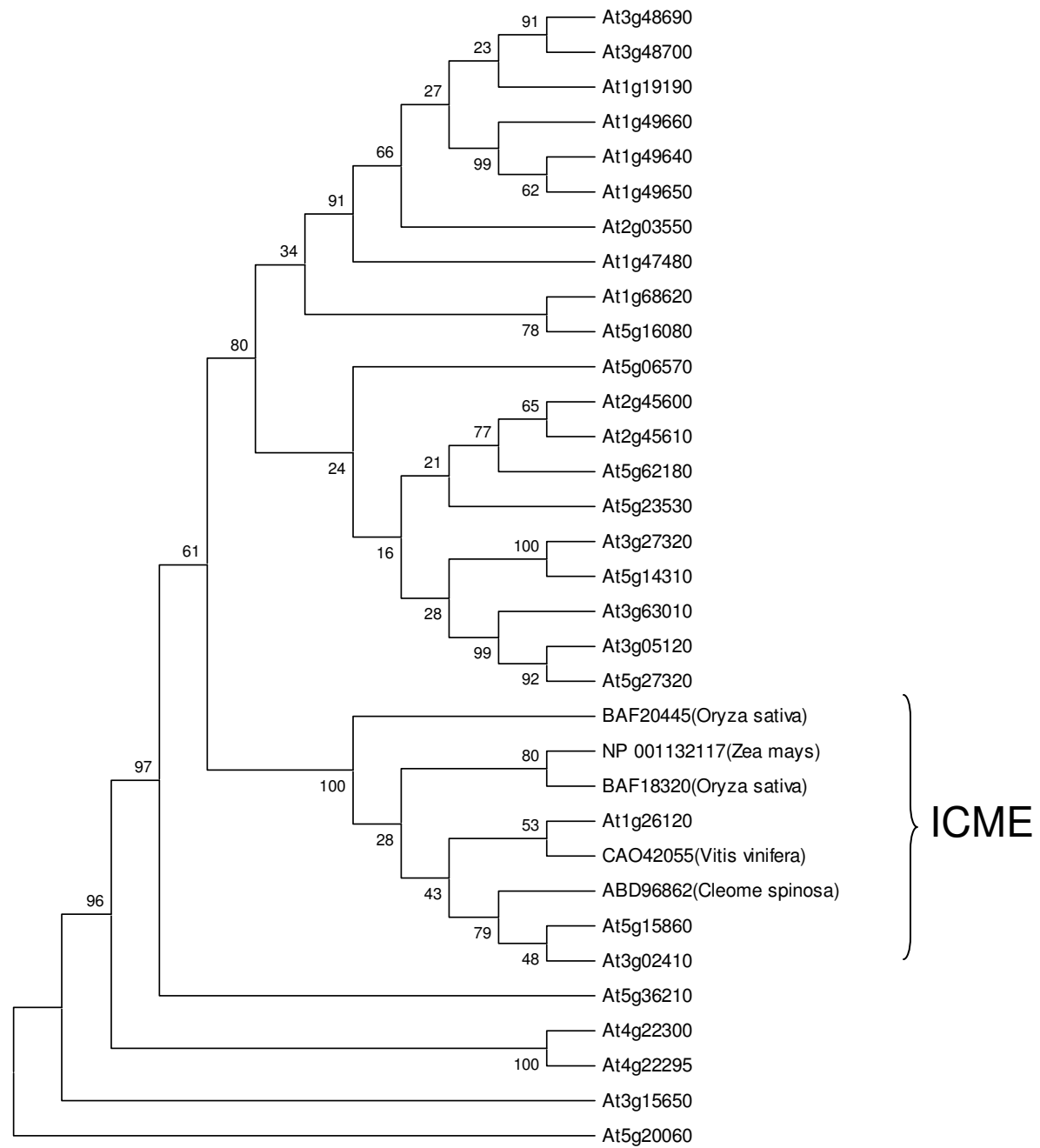

Supplement: Additional file 2 — Phylogenetic tree was constructed by Maximum Parsimony method using MEGA4 program. Phylogenetic relationships of ICME, ICME-like proteins and their homologs from Zea mays, Oryza sativa, Vitis vinifera, and Cleome spinosa as well as carboxylesterases from Arabidopsis. [file 1471-2229-10-212-S2.PDF]

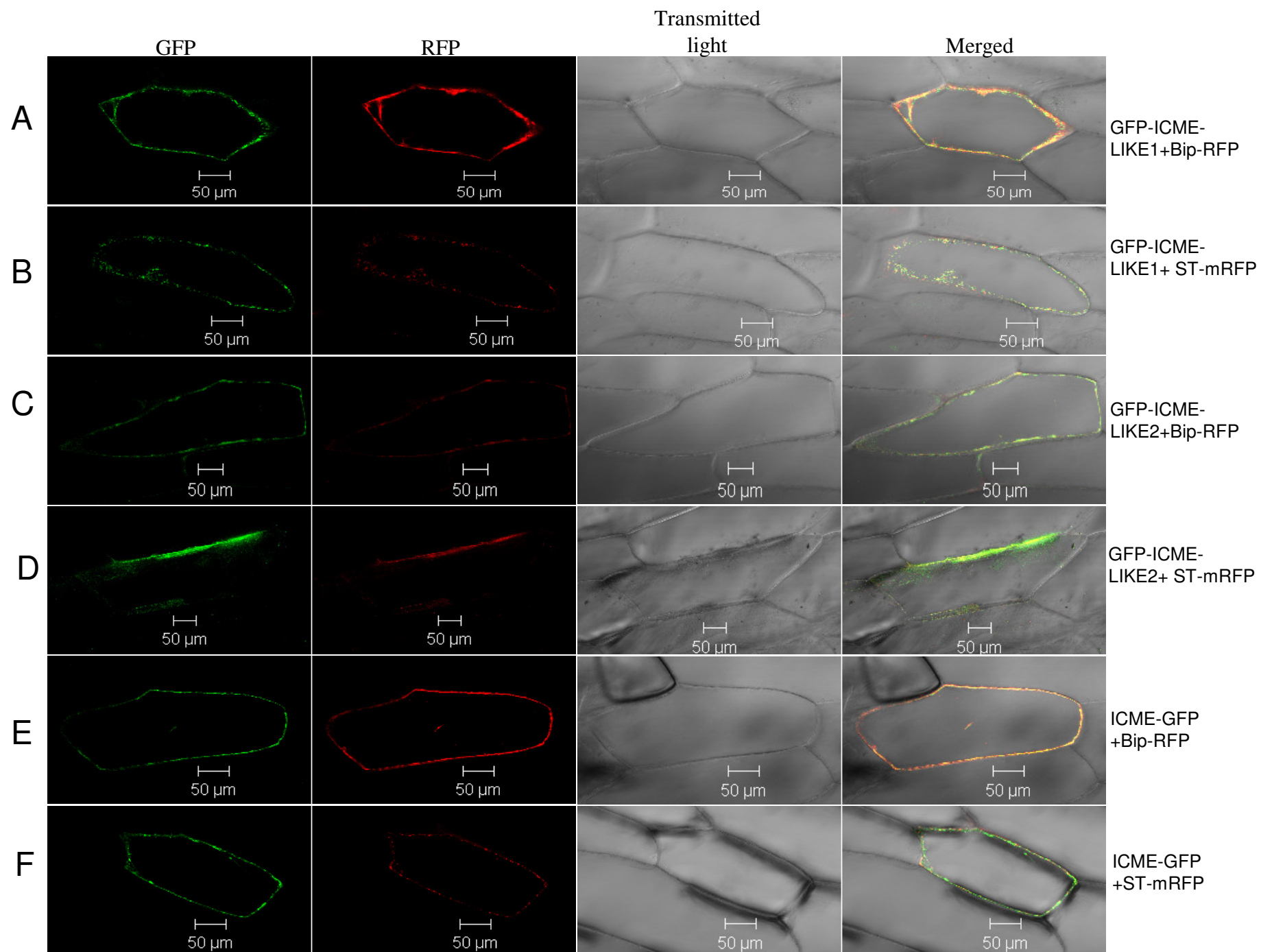

Supplement: Additional file 3 — Sub-cellular localization of GFP-tagged ICME and its homologs. Onion epidermal cells were co-transformed with free GFP, GFP-ICME-LIKE1, GFP -ICME-LIKE2, ICME-GFP and ER marker BiP-RFP (A, C and E) or Golgi apparatus marker ST-mRFP (B, D and F), respectively. For each combination shown on the right, images of GFP fluorescence, DesRed fluorescence, brightfield, and the merged were taken using a Zeiss confocal laser microscope LSM510 and were displayed from left to right, respectively. [file 1471-2229-10-212-S3.PDF]

A

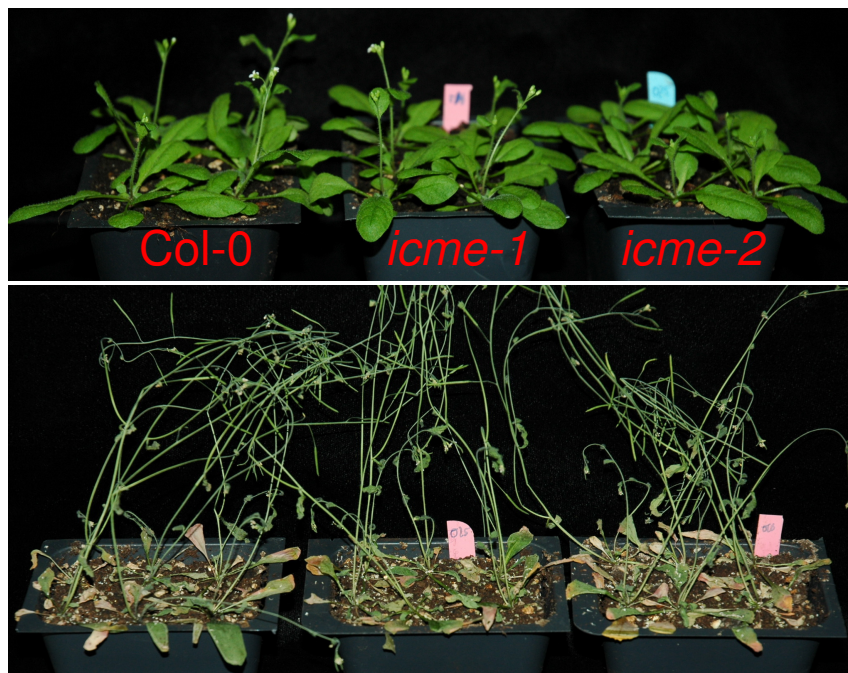

B

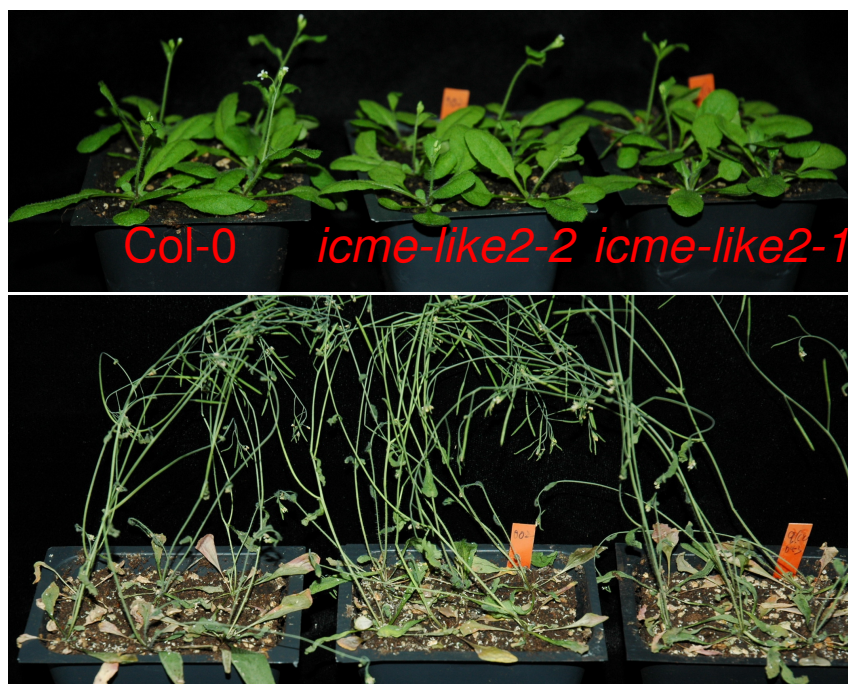

C

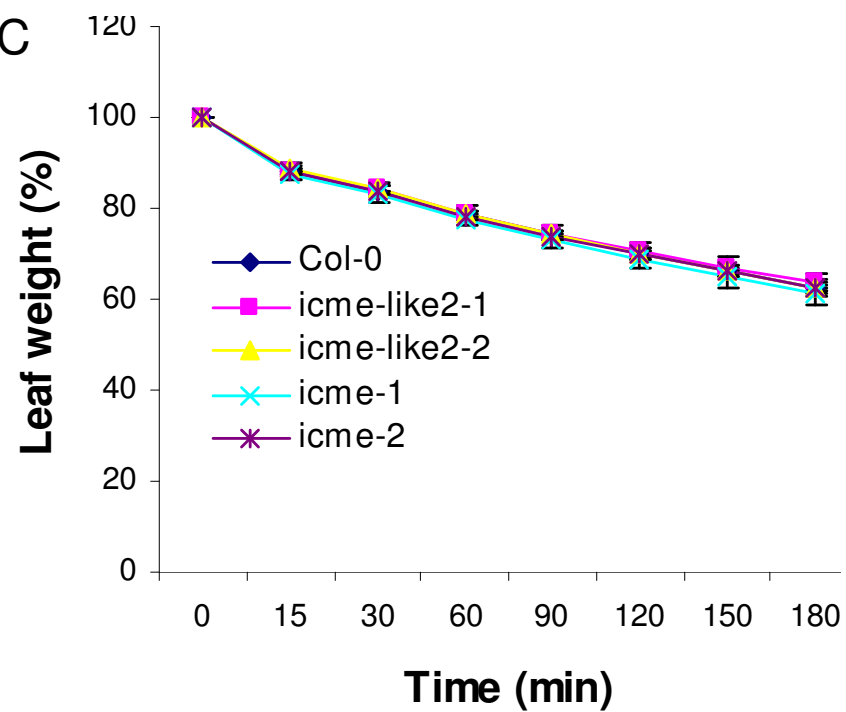

Supplement: Additional file 6 — Phenotypes, drought treatment and transpiration rates of wild type and mutants. The up panels of (A) and (B) were 21-day-old plants under normal growth condition of different genotypes. The down panels of (A) and (B) were drought treated plants. For drought treatment, 21-day-old plants under normal growth condition of different genotypes were withheld water for 14 days. (C) Transpiration rates. Rosette leaves of the same developmental stages were excised from 21-old-day plants and weighed at various time points after detachment. Each data point represents the mean of duplicate measurements. Error bars represent SD (n = 3 each). [file 1471-2229-10-212-S6.PDF]
