# Supplementary material for: Characterization, sub-cellular localization and expression profiling of the isoprenylcysteine methylesterase gene family in Arabidopsis thaliana
Source: BMC Plant Biol. 2010 Sep 27;10:212. doi: 10.1186/1471-2229-10-212 (PMC3017835; doi:10.1186/1471-2229-10-212)
Supplement: Additional file 4 — Identification of T-DNA insertion mutant lines of ICME-LIKE2 gene. (A) Gene structure of the ICME (thick lines indicate exon and thin lines indicate intron) and the locations of the T-DNA insertion in the icme-1(SALK_043448) and icme-2 (SALK_002648) lines are shown. Primers used to screen are shown as arrows. (B) Homozygous T-DNA insertion lines were identified by Genomic-PCR. icme-like2-1 was identified by two primer pairs: (1) LBa1 and gene-specific forward primer 3gRTF for confirming T-DNA insertion and (2) 3gRTIF and 3gRTR for confirming homozygous T-DNA insertion. icme-like2-2 was identified by two primer pairs: (1) LBa1 and gene-specific reverse primer 3gRTR for confirming T-DNA insertion and (2) 3gRTF and 3gRTR for confirming homozygous T-DNA insertion. (C) The flanking sequence of the T-DNA insertion lines. The position of the T-DNA insertion was confirmed by DNA sequence analysis of the resultant PCR products. Sequences underlined referred to the left border of LB, while the italic ones referred to the genomic sequences. The bold ones indicated the additional sequences due to T-DNA insertion. [file 1471-2229-10-212-S4.PDF]

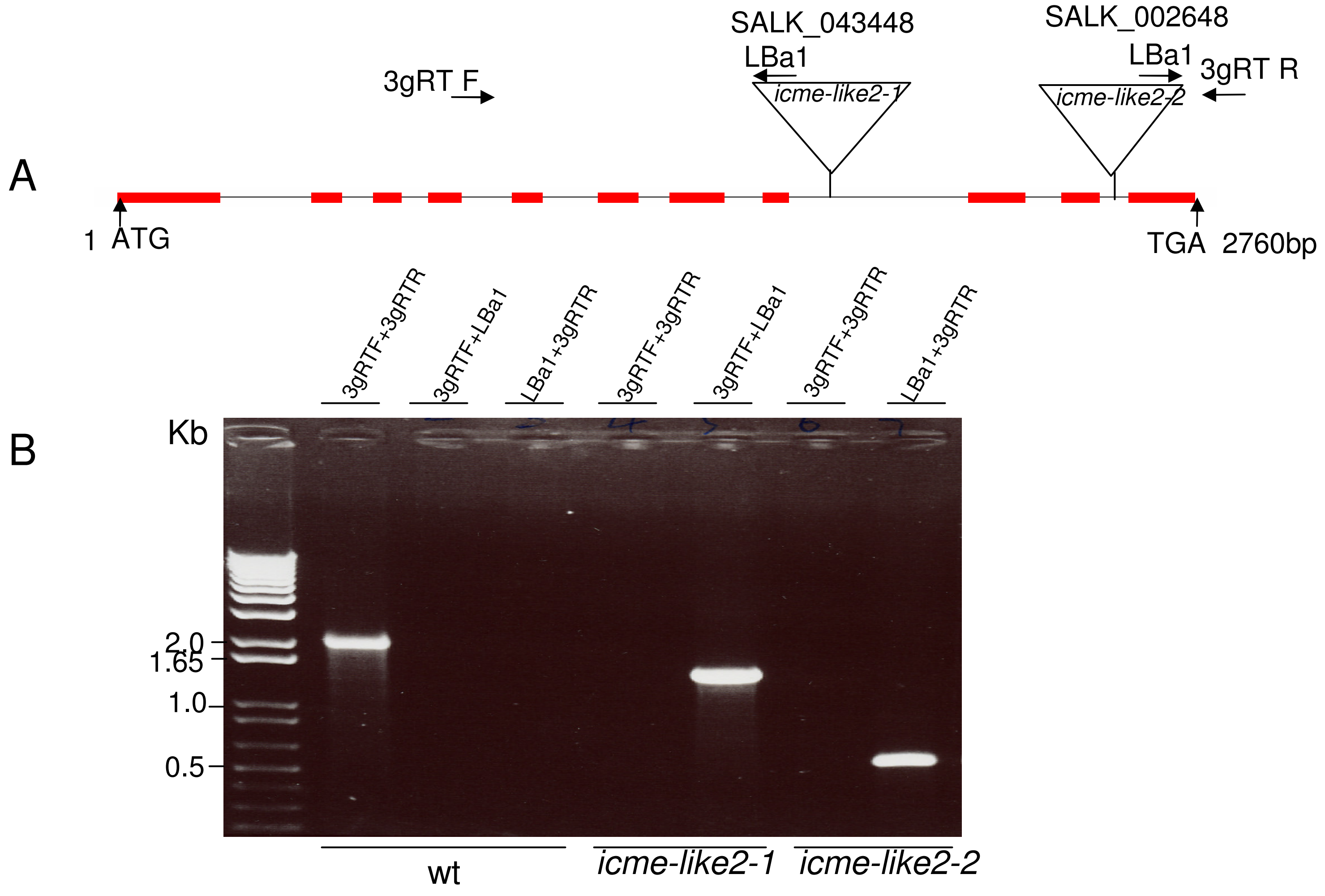


C

…*ttattatttattttttgcg*… wt

…T-DNAcaatatatcctg**tttcatgtatacatgtg***ttattatttattttttgcg*… *icme-like2-1*

…*gtctaaacacattac*… wt

…T-DNA…caatt*tgttgtctaaacacattac*… *icme-like2-2*
