# Supplementary material for: Characterization, sub-cellular localization and expression profiling of the isoprenylcysteine methylesterase gene family in Arabidopsis thaliana
Source: BMC Plant Biol. 2010 Sep 27;10:212. doi: 10.1186/1471-2229-10-212 (PMC3017835; doi:10.1186/1471-2229-10-212)
Supplement: Additional file 5 — Identification of T-DNA insertion mutant lines of ICME gene. (A) Gene structure of the ICME (thick lines indicate exon and thin lines indicate intron) and the locations of the T-DNA insertion in the icme-1(SALK_010304) and icme-2 (SALK_075701) lines are shown. Primers used to screen are shown as arrows. (B) Homozygous T-DNA insertion lines were identified by Genomic-PCR. icme-1 was identified by two primer pairs: (1) LBa1 and gene-specific forward primer 5gNcoIF for confirming T-DNA insertion and (2) 5gNcoIF and 5gRTR for confirming homozygous T-DNA insertion. icme-2 was identified by two primer pairs: (1) LBa1 and gene-specific forward primer P2 for confirming T-DNA insertion and (2) P2 and 5gRTR for confirming homozygous T-DNA insertion. (C) The flanking sequence of the T-DNA insertion lines. The position of the T-DNA insertion was confirmed by DNA sequence analysis of the resultant PCR products. Sequences underlined referred to the left border of LB, while the italic ones referred to the genomic sequence. The bold ones indicated the additional sequences due to T-DNA insertion. [file 1471-2229-10-212-S5.PDF]

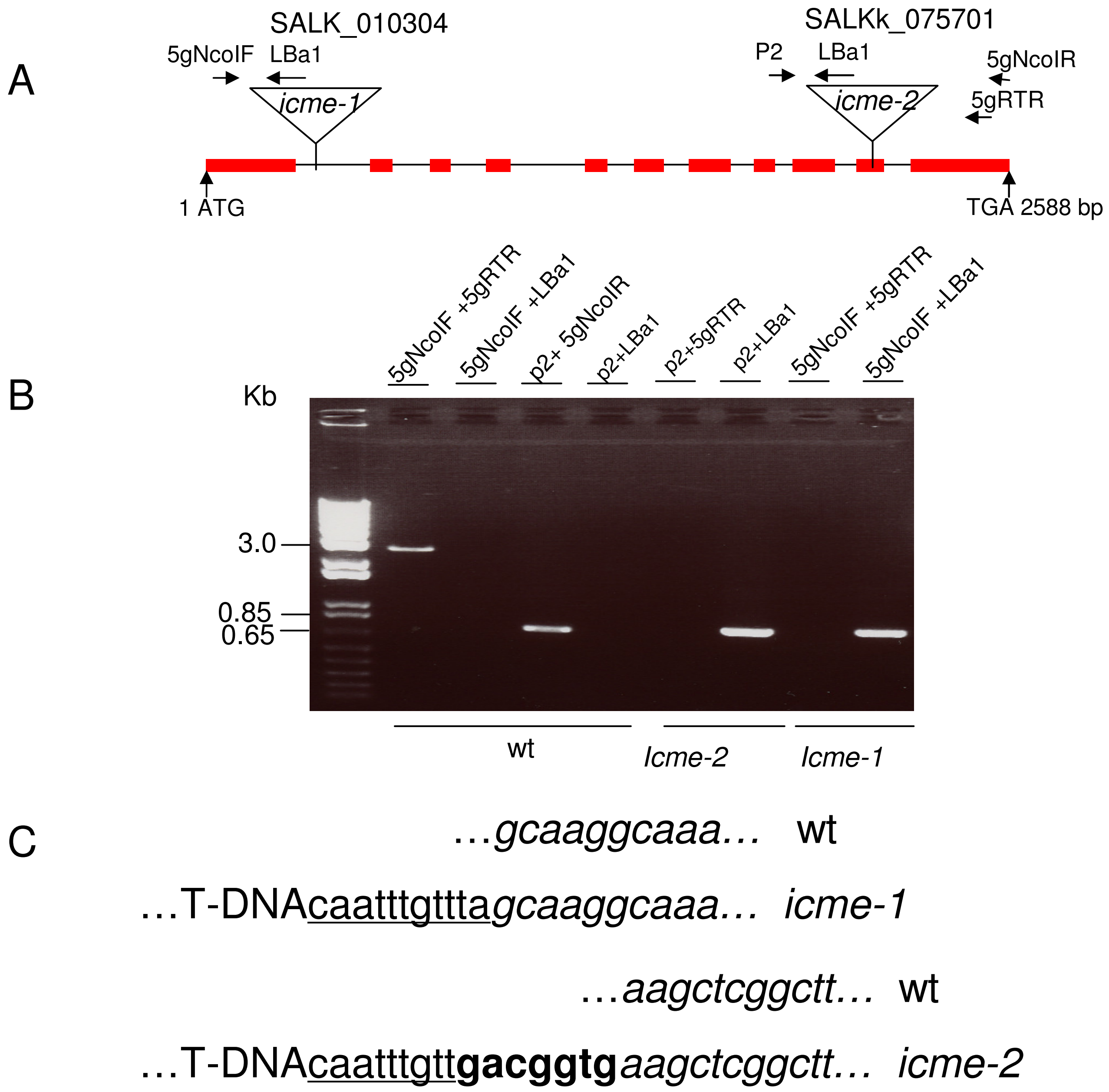
A
SALK_010304
5gNcoIF
LBa1
icme-1
SALKk_075701
P2
LBa1
icme-2
5gNcoIR
5gRTR
1 ATG
TGA 2588 bp
B
5gNcoIF +5gRTR
5gNcoIF +LBa1
p2+ 5gNcoIR
p2+LBa1
p2+5gRTR
p2+LBa1
5gNcoIF +5gRTR
5gNcoIF +LBa1
Kb
3.0
0.85
0.65
wt
Icme-2
Icme-1
C
…gcaaggcaaa… wt
…T-DNAcaatttgtttagcaaggcaaa… icme-1
…aagctcggctt… wt
…T-DNAcaatttgttgacggtgaagctcggctt… icme-2
